# A test method for selecting suitable cleaning indicators for routine cleaning monitoring on a washer-disinfector in a central sterile supply department

**DOI:** 10.1371/journal.pone.0326380

**Published:** 2025-07-01

**Authors:** Juan Zhou, Wei Guo, Dongling Liu, Jianrong Li, Caixia Yang, Ying Wang, Xiaoyi Huang

**Affiliations:** 1 Central Sterile Supply Department, Hunan Provincial People's Hospital (The First Affiliated Hospital of Hunan Normal University), Changsha, China; 2 Beijing GKE Science and Technology Co., Ltd, Beijing, China; 3 Central sterile supply department of The Second Xiangya Hospital of Central South University, Changsha, China; North-Caucasus Federal University, RUSSIAN FEDERATION

## Abstract

Cleaning indicators are widely used to evaluate the efficacy of cleaning processes in automated washer-disinfectors (AWDs) in healthcare settings. In this study, we systematically analyzed the performance of commercial indicators across multiple simulated cleaning protocols to guide the correct selection of suitable cleaning indicators in Central Sterile Supply Departments (CSSD). Eleven commercially available cleaning indicators were tested in five cleaning simulations, P0 to P4, where P1 represented the standard cleaning process in CSSD, while P2-P4 incorporated induced-error cleaning processes to mimic real-world errors. All indicators were uniformly positioned at the top level of the cleaning rack to ensure comparable exposure. Key parameters, including indicator response dynamics (e.g., wash-off sequence) and final residue results, were documented throughout the cleaning cycles. The final wash-off results given by the indicators under P0, in which no detergent was injected, were much worse than those of the other four processes. Under different simulations, the final results of the indicators and their wash-off sequences changed substantially. In conclusion, an effective indicator must be selected experimentally. The last indicator to be washed off during the normal cleaning process that can simultaneously clearly show the presence of dirt residue under induced error conditions is the optimal indicator for monitoring cleaning processes.

## Introduction

Effective cleaning is essential for removing organic and inorganic debris—including blood, tissue, and microorganisms—from the surfaces of instruments, thereby significantly reducing the microbial load before sterilization [1,2]. Notably, failure to adequately clean instruments can lead to incomplete sterilization and the potential transmission of infections between patients [3,4].

Central sterile supply departments (CSSDs) play a critical role in maintaining the efficacy and reliability of medical instrument cleaning processes regard by implementing cleaning, disinfection, and sterilization protocols. Rigorous monitoring of these processes is critical for verifying compliance, optimizing outcomes, and identifying systemic improvements [5]. Various studies have outlined rigorous cleaning monitoring methods to ensure the effectiveness of cleaning processes [6–8]. Common monitoring methods include the use of cleaning indicators simulating specific types of dirt to check residue and the application of special swabs impregnated with chemical agents that change color to indicate the presence of specific residues, such as protein or adenosine triphosphate (ATP), which are indicative of incomplete cleaning [9]. While protein or ATP swab-tests provide quantitative data, their operational complexity and labor-intensive protocols limit practical utility in high-volume CSSD workflows [10]. In contrast, cleaning indicators offer rapid, user-friendly visual feedback, making them better suited for routine monitoring in a CSSD workflow.

However, few studies have evaluated criteria for selecting proper commercial cleaning indicators that ensure optimal cleaning performance and allow for the safe release of instruments to the next step of sterilization [11,12]. The effectiveness of a cleaning indicator depends on its ability to accurately reflect the outcomes of a cleaning process, particularly under challenging conditions such as equipment malfunction, inadequate cleaning solutions, or variable water quality [13]. In this study, we address these challenges by presenting a systematic approach to selecting and evaluating cleaning monitoring indicators tailored to CSSD processes.

In this study, we implemented a testing protocol involving 11 widely used commercial indicators. These were evaluated under both **routine cleaning conditions** (simulating standard CSSD operations) and **induced-error scenarios** (e.g., detergent omission, temperature deviations) to assess their diagnostic accuracy in identifying process failures. By establishing evidence-based selection criteria, this work directly supports efforts to mitigate healthcare-associated infections and advance patient safety through robust sterilization practices.

## Materials and methods

### Materials

An automated washer-disinfector (AWD) (Marge, Shenzhen, China) in good working condition with a volume of 485 L was used in this study. Purified water with a conductivity < 15 μS/cm was used in all cleaning processes. Two detergents were used in the AWD: an enzymatic detergent, Labomat SPM (Dr. Deppe, Kempen, Germany), and an alkaline detergent, Labomat Ma Liquid (pH: 12.7; Dr. Deppe, Kempen, Germany). The recommended working temperature for the selected enzymatic detergent was between 20°C and 40°C, while that for the alkaline detergent was between 60°C and 93°C. Eleven common commercial cleaning indicators were selected and named from IA to IK: IA, Type III (Shinva, China); IB, STF (Steris, USA); IC, CDWA3 (Chemdye, Terragene, Argentina); ID, CPI Orange (GKE, Germany); IE, CDWA4 (Chemdye, Terragene, Argentina); IF, CPI Yellow (GKE, Germany); IG, CPI Green (GKE, Germany); IH, CPI Blue (GKE, Germany); II, CPI Purple (GKE, Germany); IJ, CPI Red (GKE, Germany); and IK, Wash-Checks (WC101, Getinge, Sweden). These indicators are impregnated with synthetic dirt or residual protein monitors that simulate organic dirt [12].

### Determination of cleaning indicator results

The results of the cleaning indicators were determined using numbers from 0 to 10 and letters from A to E. If the simulated dirt on the indicator was completely washed off at the end of the process, it was marked between 0 and 10, depending on the time at which the dirt had been completely washed off; this was referred to as the indicator’s wash-off sequence. The wash-off sequences were determined by analysing recorded videos of the entire cleaning process. The sequences of indicators that were washed off at almost the same time were given the same number. For repeated experiments, the median value of all recorded sequence numbers was used.

**Fig 1:**
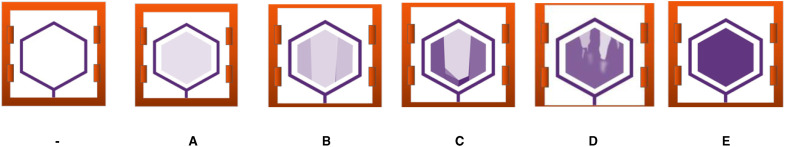
Classification of dirt left on the indicator. (-)Dirt totally washed off. (A) Small amount of dirt remaining. (B) Some amount of dirt remaining. (C) Half of the dirt remaining. (D) Most of the dirt remaining. (E) Almost appearing unwashed.

If the simulated dirt on the indicator was not washed off at the end of the process, it was marked between A and E depending on how much dirt was left on it, with A indicating more dirt having been removed and E indicating less dirt having been removed. The scale used to differentiate between indicators based on the dirt left behind is listed in Fig 1.

### Experimental methods

Only the major wash and drying stages of the AWD’s cleaning program were used in this study; the normal pre-cleaning and final rinsing were excluded, given that they have a limited effect on the indicators. There were two major wash stages. Enzymatic detergent was used in the first major wash for 5 min at 40°C, after which alkaline detergent was injected into the chamber; the second major wash then ran for 5 min at 62°C. No thermal disinfection was included in the default program.

In each experiment, all 11 indicators were placed close to each other at the edge of the highest level of the cleaning rack and facing the glass door. A camera was fixed outside the chamber toward the glass door so that during the cleaning process, the wash-off situation of the simulated dirt on the indicator surfaces could be observed and recorded through the glass door of the AWD.

To check the results of the cleaning indicators under different situations, five cleaning processes were included, labelled from P0 to P4. The cleaning programs running on the AWD for all five cleaning processes were the same. In P0, no detergent was injected. P1 was the default cleaning process in our CSSD, which employed a major enzymatic wash first followed by a major alkaline wash. P2 simulated an erroneous scenario in which the detergent bottles were improperly connected to the AWD, resulting in the alkaline detergent being used before the enzymatic detergent. In P3, only the enzymatic detergent was used, whereas in P4, only the alkaline detergent was used. Each cleaning situation was run 10 times. All experimental data, including video recordings and the final indicator results, were stored for further analysis.

## Results

In total, 50 cleaning processes were performed in this study. The differences in the final results of all indicators and their wash-off sequences varied from P0 to P4.

### Final indicator wash-off results

In P0, where no detergent was injected and the indicators were washed solely with purified water, only indicator IA was completely washed off, while ID was slightly washed off; all other indicators remained in a state almost like they had not been washed at all. In the default cleaning process P1, indicator II was the only one not completely washed off. In P2 and P4, where the enzymatic component did not function properly, indicators IF, II, and IJ showed strong resistance to the AWD’s cleaning performance, either washing off late in the cleaning process or being incompletely washed off. In P3, where only the enzymatic detergent worked, indicator IF was washed off quite early, while IG and IH showed much higher resistance than in the alkaline environment, washing off later or incompletely. All final wash-off results are illustrated in Table 1.

**Table 1:** Final wash-off results for all indicators.

Program	IA	IB	IC	ID	IE	IF	IG	IH	II	IJ	IK
P0	–	D	C	A	D	E	E	E	E	E	–
P1	–	–	–	–	–	–	–	–	A	–	–
P2	–	–	–	–	–	–	–	B	D	E	–
P3	–	–	–	–	–	E	E	–	E	E	–
P4	–	–	–	–	–	–	–	E	C	E	–

IA to IK: 11 indicators, P0 to P4: 5 programs, A to E: different dirt levels left on the indicator after the washing process, -: completely washed off after the cleaning process.

### Wash-off sequence of indicators that were completely cleaned

Indicator IA was always the first to be washed off in all processes, including P0, where no detergent was used. Indicators IB, IC, and ID were also washed off easily in P1 through P4. Indicators IE, IF, and IG showed very similar wash-off resistance in the default cleaning process P1 and the simulated error processes P2 and P4. P1 was the only process that could remove dirt from indicator IJ. The wash-off sequence of indicator IK could not be identified due to the poor contrast of its dirt against the metal carrier material. All wash-off sequences were listed in Table 2.

**Table 2:** Wash-off sequence of the indicators.

Program	IA	IB	IC	ID	IE	IF	IG	IH	II	IJ	IK
P0	0	–	–	–	–	–	–	–	–	–	*
P1	0	0	1	1	3	3	3	2	–	4	*
P2	0	0	1	1	3	2	3	–	–	–	*
P3	0	1	2	2	4	–	–	3	–	–	*
P4	0	1	2	2	4	3	4	–	–	–	*

IA to IK: 11 indicators, P0 to P4: 5 programs, 0–10: wash-off sequence of the indicators (median value), -: incompletely washed off.

## Discussion

The removal of organic and inorganic residues from instrument surfaces by effective cleaning not only mitigates the risk of healthcare-associated infections, but also plays a pivotal role in optimizing the sterilization process [7,14]. Thorough cleaning ensures that sterilization agents could effectively penetrate and eradicate microorganisms, thus reducing the potential for surgical site infections [15]. Moreover, pristine instrument surfaces enhance the functionality and longevity of medical devices, safeguarding against equipment malfunction or adverse patient outcomes during critical procedures [16]. Therefore, investing in comprehensive cleaning protocols is essential for healthcare facilities to enhance procedural outcomes.

The current landscape of cleaning monitoring requirements and methods within various standards reflects a growing emphasis on ensuring the efficacy and consistency of cleaning processes in healthcare facilities. Standards such as those established by ISO and AAMI outline stringent guidelines for monitoring cleaning to mitigate potential risks [17,18]. These standards typically advocate a multifaceted approach to monitoring, encompassing visual inspection, chemical and biological indicators, and technological advancements such as fluorescence-based inspection systems [10]. However, despite the availability of comprehensive guidelines, challenges persist in achieving uniform compliance. Potential risks include inadequate training of personnel, improper selection or use of detergents, and the potential for human error or oversight [9]. Addressing these challenges requires ongoing collaboration among regulatory bodies, healthcare professionals, and industry stakeholders to foster a culture of accountability, and best practices in cleaning monitoring protocols [4,13].

The Sinner’s Circle, also known as the four parameters of cleaning, is a fundamental concept for understanding the cleaning process [2,8]. The four parameters, namely, mechanical action, time, temperature, and chemical action, are interconnected factors that influence cleaning efficiency. Mechanical action refers to the physical force applied during cleaning, which helps to dislodge soil from surfaces; time represents the duration of the cleaning process; temperature affects the speed and efficacy of the chemical reactions involved in cleaning; and chemical action involves the interaction between cleaning agents and contaminants to break down and solubilize soils. This framework underscores that deviations in any parameter can compromise overall performance, while adjustments to one factor may necessitate recalibration of others to maintain effectiveness. A critical limitation of the model is its inherent complexity: diminished cleaning outcomes rarely stem from isolated parameter failures but rather from dynamic interactions among variables. This represents the complexity of cleaning processes, and our experimental design was based on the theory of the Sinner’s Circle.

In this study, 11 common commercial cleaning indicators were tested to determine how their response varies when factors in cleaning processes change. The aim of this study was to identify a means to correctly select suitable cleaning indicators for routine work undertaken in a CSSD. All indicators were placed at similar positions inside one AWD running the same program. The default program includes two major wash stages, including enzymatic detergent and alkaline detergent, to maximize the cleaning effect. Different scenarios were employed to simulate mistakes that have the potential to occur in a CSSD during daily operation. The final status of each indicator was photographed, and the entire cleaning process was recorded in a video.

a) P0: Control group (only washed with purified water)
b) P1: Default cleaning procedure (enzymatic detergent first, then alkaline detergent)
c) P2: Incorrect/reversed connection (alkaline detergent first, then enzymatic detergent)
d) P3: Incorrect injection procedure (enzymatic detergent alone)
e) P4: Incorrect injection procedure (alkaline detergent alone)

First, by comparing the results of the control group to those of other experimental groups, we found that regardless of any changes in the cleaning process, as long as a detergent is used in the process, there is a significant enhancement in the overall removal of dirt on indicators. While our model system utilized synthetic contaminants rather than actual contaminants on real instruments, these controlled experiments provide mechanistic evidence supporting detergent’s essential role in automated cleaning processes. We also found that some indicators can always be removed even with only water, as demonstrated by our results in the control group P0. Conversely, some indicators cannot be removed regardless of any cleaning processes employed. It is apparent that these two types of indicators are unsuitable for monitoring the cleaning process discussed in this study because they have a resistance to cleaning that is either too low or too high.

The default cleaning process used in this study involved sequential application of enzymatic detergent followed by alkaline detergent. Comparative analysis of three simulated induced-error cleaning processes revealed substantial variations in the final cleaning results observed for all indicators. This outcome was in line with predictions derived from the Sinner’s Circle theory, where intentional modifications to key process parameters significantly impacted cleaning performance. Intriguingly, indicator wash-off sequence exhibited process-dependent variability. Specific cases demonstrated divergent behaviours–one indicator could be rapidly cleaned in one process but remained nearly unchanged in another (e.g., indicator IH was cleaned quickly in P1 but was almost like unwashed in P4). We attribute this phenomenon to the different chemical interactions occurring between the detergent components and contaminants. The enzymatic components in the multi-enzyme detergent may react differently to a specific contaminant in the highly alkaline environment created by the alkaline detergent. Enzymatic detergents contain specific enzymes (e.g., proteases and amylases) that act as catalysts to accelerate the breakdown of complex organic molecules into smaller, more soluble fragments. Alkaline detergents utilize high-pH solutions to emulsify and solubilize a broad range of contaminants, including fats, oils, and mineral deposits [19]. It is precisely because of this discovery that different indicators have different wash-off sequences under different conditions that relying solely on the final cleaning result to select appropriate cleaning indicators seems overly simplistic.

Given the discovery of varying sequences in which indicators are cleaned under different washing conditions, for a specific cleaning process, the indicator that is cleaned the last among all the cleaning indicators is likely the most suitable for monitoring this process. This is because it monitors the greatest accumulation of total cleaning efficacy. Ideally, the most suitable indicator should be cleaned in the last minute of the major wash process. As soon as this particular cleaning process becomes less effective, operators can immediately make judgments based on the uncleaned status of this indicator. In other words, by identifying the last indicator to be cleaned in a specific cleaning process through testing, monitoring objectives can be achieved in future cleaning processes using this indicator for each batch.

However, based on the experimental data, we also encountered issues with this method of assessment. For instance, while indicators IE and IG were cleaned relatively late in the default process, they could still be cleaned in the erroneous washing processes P2 and P4. This implies that even in the event of serious detergent errors during practical operations, these indicators cannot signal the occurrence of an error. Therefore, the correct indicator should not only be those that are cleaned later in the default cleaning process, but that also exhibit significant differences when several types of common errors occur. This difference is exemplified in indicator IJ, which is cleaned last in the default procedure and is almost impossible to clean in all error processes. Consequently, for the specific cleaning process discussed in this study, IJ is the most suitable indicator for monitoring.

The experimental results also demonstrate that there is no universally effective cleaning indicator. If a cleaning indicator is chosen without conducting any experiments and is used directly, it will inevitably lead to one of two problems: (1) this indicator might be very easily cleaned in the specific process used by the CSSD, rendering it meaningless for monitoring; (2) this indicator cannot be cleaned at all even when the program and machine are functioning normally. In these cases, many operators may believe that the detergent’s efficacy is inadequate. However, this inference is flawed because there may be no direct logical connection between whether an indicator is cleaned and whether the medical instruments are cleaned. Indeed, it is possible that the instruments have already been sufficiently cleaned while an indicator remains uncleaned, or conversely. Cleaning indicators can only serve as monitoring tools for the cleaning process, and not for determining the cleanliness of the instruments themselves. Therefore, validation is essential for linking these two concepts.

The underlying principle of selection is that multiple experiments must be conducted to identify an indicator that is the last to be cleaned in a specific cleaning process in a CSSD, while also exhibiting significant differences in some error processes. Incomplete experimental results render the use of indicators meaningless. Based on the experimental results, we propose the following recommendations for correctly selecting cleaning monitoring indicators in CSSDs.

1. Use various cleaning indicators in different tests as much as possible. Ensure that the indicators are placed close to each other on the top layer inside the chamber so that they are in positions that are more difficult to reach during cleaning. Additionally, ensure that the indicators face toward the glass window of the wash disinfector, allowing the operators to record the entire process from outside the window.
2. Use the default cleaning program of the CSSD and meticulously record the cleaning sequence of the indicators throughout the entire process. Record the final results after completion.
3. Design and run several processes with significant errors, such as no addition of detergent, insufficient addition of cleaning solution, excessively high/low temperature during major washes, and poor water quality. During the execution of the error processes, place the indicators in the same positions and record the entire process through the glass window.
4. Select the indicators that are cleaned last in the default program and examine whether they show uncleaned results during the error processes. In case of uncleaned results, these indicators can be considered the best monitoring indicators for this specific cleaning process in the CSSD; otherwise, look for indicators that are cleaned second to last in the default program.

To ensure consistency, we recommend repeating the aforementioned procedures at least three times. Special attention should be paid to whether the suction tubes contain residues from the previous cleaning program, as these detergent residues can greatly affect the results of the next experiment. Flushing the suction tubes or running an empty cycle before each experiment is recommended.

Through these experiments, CSSD operators can effectively identify the most suitable cleaning indicator product from the multitude of products available in the market for their specific cleaning process. This enables effective monitoring of the stability of each batch of cleaning processes. It is important to note that the experiment must be repeated whenever important variables in a specific cleaning process change, such as by changing the cleaning solution, replacing the automatic washing machine, or establishing a new CSSD.

## Conclusions

This study focuses on the selection of appropriate cleaning monitoring indicators tailored to specific processes within CSSDs. Multiple experiments were conducted using diverse cleaning indicators strategically positioned in the cleaning chamber and oriented toward a glass window for external video recording. After discovering that different cleaning processes could not only influence the final cleaning result on indicators but also their wash-off sequence, we introduced a methodology for selecting appropriate cleaning indicators. Correct selection could only be performed by multiple experiments, including normal cleaning processes and some cleaning processes with obvious errors, such as using an inadequate detergent and extreme temperatures. By following this rigorous methodology, CSSD operators can effectively identify the most suitable cleaning indicators for their specific processes, enabling consistency among batches of cleaning processes. It is important to emphasize that whenever key variables in the cleaning process change (e.g., new cleaning solutions, equipment, or CSSD establishment), the experiments should be repeated to confirm the performance of the indicator under the new conditions.

## Supporting information

S1 Data Raw Data. (XLSX)
